# Prefrontal modulation of anxiety through a lens of noradrenergic signaling

**DOI:** 10.3389/fnsys.2023.1173326

**Published:** 2023-04-17

**Authors:** Nadia N. Bouras, Nancy R. Mack, Wen-Jun Gao

**Affiliations:** Department of Neurobiology and Anatomy, Drexel University College of Medicine, Philadelphia, PA, United States

**Keywords:** prefrontal cortex, locus coeruleus, anxiety, norepinephrine, noradrenaline, noradrenergic receptors, stress

## Abstract

Anxiety disorders are the most common class of mental illness in the U.S., affecting 40 million individuals annually. Anxiety is an adaptive response to a stressful or unpredictable life event. Though evolutionarily thought to aid in survival, excess intensity or duration of anxiogenic response can lead to a plethora of adverse symptoms and cognitive dysfunction. A wealth of data has implicated the medial prefrontal cortex (mPFC) in the regulation of anxiety. Norepinephrine (NE) is a crucial neuromodulator of arousal and vigilance believed to be responsible for many of the symptoms of anxiety disorders. NE is synthesized in the locus coeruleus (LC), which sends major noradrenergic inputs to the mPFC. Given the unique properties of LC-mPFC connections and the heterogeneous subpopulation of prefrontal neurons known to be involved in regulating anxiety-like behaviors, NE likely modulates PFC function in a cell-type and circuit-specific manner. In working memory and stress response, NE follows an inverted-U model, where an overly high or low release of NE is associated with sub-optimal neural functioning. In contrast, based on current literature review of the individual contributions of NE and the PFC in anxiety disorders, we propose a model of NE level- and adrenergic receptor-dependent, circuit-specific NE-PFC modulation of anxiety disorders. Further, the advent of new techniques to measure NE in the PFC with unprecedented spatial and temporal resolution will significantly help us understand how NE modulates PFC function in anxiety disorders.

## Introduction

Anxiety is defined as the anticipation of future threat (American Psychiatric Association [APA], 2013). This physiological and psychological response is thought to be a normal, healthy, adaptive response to aid in survival in an ever-changing world. However, persistent, disruptive, and exacerbated anxiety can become debilitating through threat-generalization to non-threatening situations, producing a constant state of heightened arousal. Pathological anxiety disorders are separated into three main categories: obsessive-compulsive and related disorders, trauma- and stressor-related disorders, and generalized anxiety disorders (American Psychiatric Association [APA], 2013). Although these disorders vary in their etiology, in all cases, the resulting cognitive and behavioral deficits significantly impair normal functioning. Not only do disorders of this nature affect an individual’s performance at school/work, relationships, and self-esteem, but they also lead to significant economic and personal burdens (Bereza et al., 2009; Mondin et al., 2013; Pagotto et al., 2015). Anxiety disorders have a lifetime prevalence of 28% (Kessler et al., 2005), affecting about 40 million individuals in the United States of America and 970 million worldwide. Despite the commonality of these disorders, generalized anxiety disorder (GAD) is one of the least successfully treated psychiatric disorders (Li et al., 2020), and progress toward anxiolytic drug discovery has been slow (Griebel and Holmes, 2013). The treatment gaps in GAD and other anxiety disorders result from our limited understanding of the biological mechanisms by which anxiety symptoms emerge or how these mechanisms are altered by current treatments (Li et al., 2020).

It is increasingly recognized that cognitive deficits underlie various symptoms associated with stress-related psychiatric illnesses, such as anxiety (Beck, 2005; Moran, 2016). A frontal brain structure heavily involved in cognitive functioning is the prefrontal cortex (PFC). This brain region exerts top-down control over behavior, thought, and emotion (Datta and Arnsten, 2019). Lesions of the PFC produce symptoms such as poor judgment, increased distractibility and hyperactivity, poor attentional regulation, and disorganized behavior (Arnsten, 1998), similar to the symptoms seen in anxiety disorders. This suggests the PFC may be implicated in the pathophysiology of anxiety (Kenwood et al., 2022).

**FIGURE 1:**
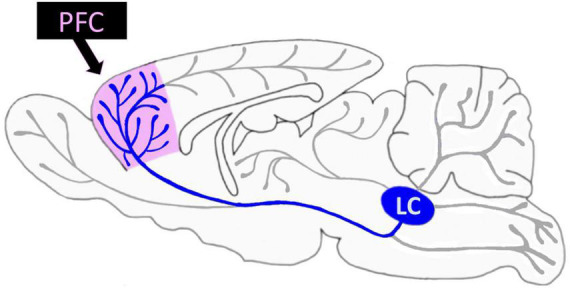
Rodent locus coeruleus (LC) projections are widespread but especially dense in the prefrontal cortex (PFC). The LC projects widely across the rodent brain (gray) including the PFC (pink); LC-PFC projections are particularly dense (blue) compared to other LC projections.

**FIGURE 2:**
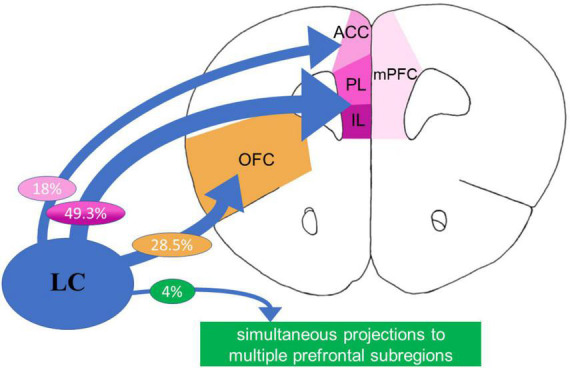
The rodent prefrontal cortex (PFC) receives dense projections from the locus coeruleus (LC). Subregions of the rat medial prefrontal cortex (mPFC) receive dense innervation from the LC, with significantly higher amount of norepinephrine (NE) varicosities (Agster et al., 2013) and high percentage of LC projections to the prelimbic (PL)/infralimbic (IL) regions compared to other cortical regions, and relatively low (4%) overlap (Chandler et al., 2014a).

One neurotransmitter that is thought to play an extensive role in both anxiety and modulation of PFC function is norepinephrine (NE). The locus coeruleus (LC), a brainstem structure, provides the primary source of NE to the mammalian neocortex (Chandler et al., 2014b; Poe et al., 2020; Breton-Provencher et al., 2021; Ross and Van Bockstaele, 2021; see Figures 1, 2). Cortical projections from the LC are heterogeneous, with distinct biochemical and electrophysiological properties (Chandler and Waterhouse, 2012; Chandler et al., 2014a; Morris et al., 2020). Further, these minimally divergent projection neurons coordinate their molecular phenotypes and physiological profiles to the operation of their specific terminal fields, governing varying levels of NE release. For example, the LC projects to the PFC with much denser NE varicosities compared to other cortical regions such as sensory and motor cortices (Agster et al., 2013). This unique arrangement makes sense in terms of behavioral significance, since the LC exhibits more robust modulatory actions (such as greater NE release) in prefrontal decision-making circuits compared to circuits related to motor movement.

Recent research has indicated that both NE and the PFC are extensively involved in anxiety etiology through distinctly different cell-type, microcircuit-, and macrocircuit-level modulation (Goddard et al., 2010). However, how NE modulation in the PFC coordinates action to optimize PFC function for appropriate attention, cognition, and behavior, and how this may go awry in pathological anxiety states remains unclear. This review summarizes current rodent, primate, and human literature regarding the neurobiology of LC-NE-PFC regulation. Further, we will bridge the work between what is known about LC-NE modulation in pathological anxiety and what is known about prefrontal regulation of pathological anxiety. We hope to shed light on the many remaining unknowns, which may be important for improving the therapeutic arsenal for the management of anxiety disorders.

## LC-NE system modulates PFC neural activity

As introduced above, the PFC receives uniquely dense innervation from the LC, surpassing the degree of NE varicosities in other crucial brain sensory regions, including, motor, and thalamic regions (Agster et al., 2013; Figures 1, 2). It is proposed that activation of the LC and subsequent NE release terminates the brain’s resting state and commences a brain-state adjustment to orchestrate attention (Corbetta et al., 2008; Sestieri et al., 2011; Tang et al., 2012; Buckner, 2013), facilitate task-relevant behaviors, and help optimize task performance (Aston-Jones and Cohen, 2005). Investigators found that both within and between trials, LC neuron depolarization occurs before forebrain neural activity and is related to cognition (Bouret and Sara, 2005). LC-released NE has a robust effect on the functional integrity of the PFC. As LC-NE neuronal firing rate is associated with the attentional state, it has long been appreciated that NE significantly affects various attentional processes governed by the PFC. NE modulates cortical function during vigilance, attention, arousal, and stress (Aston-Jones et al., 1991; Berridge et al., 1993; Berridge and Waterhouse, 2003; Morilak et al., 2005). Specifically, noradrenergic signaling in the PFC is essential for cognitive changes associated with each of these states (Aston-Jones et al., 2000) and plays a modulatory role in the higher order functioning required to adapt to the demands of a changing or stressful environment (Lapiz and Morilak, 2006; Bondi et al., 2010; Arnsten, 2015).

Though differential innervation of the mPFC subregions (Chandler et al., 2013; Cerpa et al., 2019) and functional disassociations in these subregions (Cerpa et al., 2019) is recognized, the distinct modulatory effects of NE on the ACC, PL and IL prefrontal subregions remain barely studied. Nevertheless, some reported data indicate potential differential modulatory effects of NE on the PFC in a subregion-specific manner. For example, a recent study investigating changes in the norepinephrine transporter (NET) and dopamine-beta-hydroxylase (DBH) density in functionally distinct subregions of the PFC, including IL, PL, ACC, and OFC in adolescent rats found that NET, but not DBH, changed across adolescence in a regionally selective manner. The PL and the OFC showed higher levels of NET at early adolescence (Bradshaw et al., 2016). Additionally, infusion of methylphenidate, an NET inhibitor, into the ACC and IL, but not PL and OFC, inhibited social play (Achterberg et al., 2015), indicating NE-mediated region-specific inhibitory effects (Achterberg et al., 2015). In addition, the mPFC output to subcortical brain areas is known to control different cognitive, social, and emotional processing. Beyond these studies, however, it remains unknown whether and how NE may play unique modulatory roles in distinct subregions and cell types of the PFC. Also, despite the known effects of NE on general PFC-dependent cognition and attention and its interactions with stress, the spatiotemporal dynamic of adrenergic modulation of PFC-dependent behavior remains elusive (Breton-Provencher and Sur, 2019; Breton-Provencher et al., 2021, 2022). Thus, more research is needed to understand the differential and subregion-specific PFC-NE mechanisms associated with anxiety-like behaviors. We will first examine what is known about NE modulation at the molecular level in the PFC, how this affects behaviors, and what remains to be explored.

### Laminar and synaptic distribution of adrenergic receptors

Norepinephrine modulates neural activity through various types of adrenergic receptors (ARs, Box 1). All subdivisions of the PFC contain cells expressing one or more variations of α- or β-adrenergic receptors and subtypes. The various AR receptors have been identified in both excitatory and inhibitory PFC neurons across numerous cortical layers pre- and post-synaptically (Tables 1, 2).

**BOX 1 Noradrenergic receptor overview.**

Noradrenergic responsivity is mediated by three adrenergic receptors (ARs) in the brain: α1, α2, and β adrenergic receptors. Each family of these different G-protein-coupled receptors plays a distinct, often opposing, role in the brain based on their intrinsic signaling pathways.

 α1 receptors (consisting of three sub-types: α1A, α1B, and α1D) display anatomic and functional differences throughout the PFC depending on the receptor subtype. α1 receptors signal via the Gq-protein coupled receptor cascade, where they are coupled to the PKC signaling pathway. PKC signaling is mediated through activation of phospholipase CàDAG pathway, generating DAG and IP3. IP3 stimulates the release of intracellular Ca2+. Previous research has shown post-synaptic α1 receptor activation in the PFC may disengage optimal prefrontal functioning, as shown through impaired working-memory performance.

 α2 receptors (consisting of three sub-types: α2A, α2B, and α2D) signal through the Gi-protein coupled receptor cascade. Of the three subtypes, α2A is overwhelmingly predominant in the PFC. Following activation of α2 receptors, cAMP production is inhibited, which in turn, inhibits PKA and prevents phosphorylation of downstream proteins. In addition, inhibition of cAMP production reduces cAMPmediated opening of K+ channels and inhibits HCN channels. Closure of HCN channels on PFC dendritic spines suppresses isolated excitatory inputs and enhances responses to coherent bursts of synaptic activity, resulting in increased synaptic efficacy between communicating neurons. Additional activation of α2A receptors colocalized with HCN channels participate in signal enhancement and consequent improvements in the network “signal-to-noise” ratio through Gi-mediated inhibition of cAMP. Previous research has shown post-synaptic α2 receptor activation in the PFC may engage prefrontal functioning, as shown through enhanced working-memory performance. Contrastingly, presynaptic α2 noradrenergic receptors serve as autoreceptors and participate in a noradrenergic negative feedback mechanism to promote the closure of Ca2+ channels on NE axons, eventually inhibiting NE release in the synapse.

 β receptors (consisting of three sub-types: β1, β2, and β3) signal through the Gs-protein coupled receptor cascade. Following activation of β receptors, adenylyl cyclase initiates a cAMP-dependent protein kinase A (PKA) activation, resulting in the phosphorylation of Ca2+ channels and an increase in Ca2+ influx, thus, exciting pre-synaptic neurons and enhancing NE release in the synapse. Both pre- and post-synaptic β-ARs in layer V/VI mPFC pyramidal neurons enhance excitatory neurotransmission, though effects of these receptors, especially post-synaptically, have yet to be specifically studied in other distinct mPFC layers.

**TABLE 1A:** Laminar and cellular distribution of α-adrenergic receptors (AR) in the medial prefrontal cortex (mPFC).

A) Laminar distribution of different α -adrenergic receptors subtypes in medial prefrontal cortex (mPFC) layers I–VI.					
	**mPFC layer**				
**α -AR receptor subtype**	**I**	**II/III**	**V**	**VI**	**References**
α1A	**–**	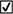	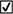	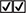	Marek and Aghajanian, 1999; Santana and Artigas, 2017; Santana et al., 2013
α1B	**–**	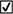	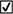	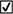	Marek and Aghajanian, 1999; Santana and Artigas, 2017; Santana et al., 2013
α1D	**–**	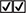	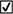	**–**	Marek and Aghajanian, 1999; Santana and Artigas, 2017; Santana et al., 2013
α2A	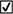	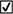	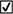	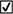	Goldman-Rakic et al., 1990; Ramos and Arnsten, 2007
α2B	Below the threshold for detection				Aoki et al., 1998a
α2C	Below the threshold for detection				Aoki et al., 1998a
**B) Presence of α -adrenergic receptor subtypes on excitatory and inhibitory neurons in the medial prefrontal cortex (mPFC).**					
	**Type of mPFC neuron**		**Synaptic location**		
**α -AR receptor subtype**	**Glutamatergic (excitatory)**	**GABAergic (inhibitory)**	**Pre-synaptic**	**Post-synaptic**	**References**
α1A	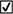	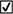	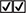	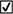	Berridge, 2008; Mitrano et al., 2012; Santana et al., 2013; Santana and Artigas, 2017
α1B	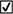	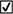	?	?	Achterberg et al., 2015; Bradshaw et al., 2016
α1D	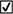	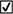 (sparsely)	?	?	Achterberg et al., 2015; Bradshaw et al., 2016
α2A	?	?	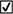	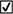	Berridge and Waterhouse, 2003; Brocos-Mosquera et al., 2021
α2B	?	?	?	?	–
α2C	?	?	?	?	–

**TABLE 2A:** Laminar and cellular distribution of β-adrenergic receptors in the medial prefrontal cortex (mPFC).

A) Laminar distribution of of different β -adrenergic receptor subtypes in medial prefrontal cortex (mPFC) layers I–VI.					
	**mPFC layer**				
**β -AR receptor subtype**	**I**	**II/III**	**V**	**VI**	**References**
β1	**–**	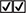	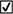	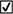	Liu et al., 2014
β2	**–**	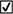	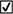	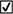	Goldman-Rakic et al., 1990; Zhou et al., 2013
β3	?	?	?	?	**–**
**B) Presence of β -adrenergic receptor subtypes on excitatory and inhibitory neurons in the medial prefrontal cortex (mPFC).**					
	**Type of mPFC neuron**		**Synaptic location**		
**β -AR receptor subtype**	**Glutamatergic (excitatory)**	**GABAergic (inhibitory)**	**Pre-synaptic**	**Post-synaptic**	**References**
β1	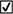	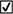	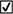	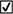	Aoki et al., 1998b; Ji et al., 2008; Torkaman-Boutorabi et al., 2014
β2	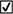	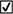	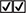	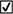	Aoki et al., 1998b; Ji et al., 2008; Zhou et al., 2013
β3	?	?	?	?	**–**

### Behavioral implications of adrenergic receptors

**FIGURE 3:**
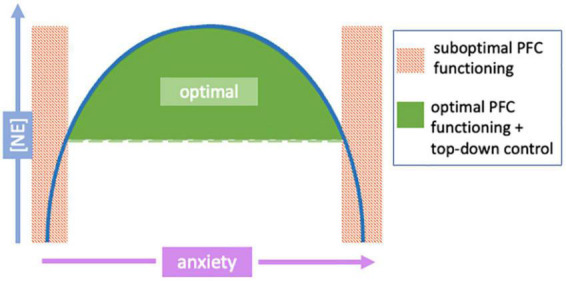
The relationship between norepinephrine (NE) levels and anxiety follows an inverted-U shaped correlation. The relationship of NE release and anxiety levels follows an inverted-U shape. Green areas indicate moderate and manageable arousal/anxiety levels as a result of moderate NE release; this area of a “happy medium” allows continued optimal prefrontal cortex (PFC) functioning and top-down control (and thus, normal behavior). Red shaded areas show areas of either hyper- or hypo-NE release, causing the PFC to be taken “offline” causing loss of necessary regulatory control over other brain regions (impaired PFC-dependent functions). Modified from Arnsten (2011).

**FIGURE 4:**
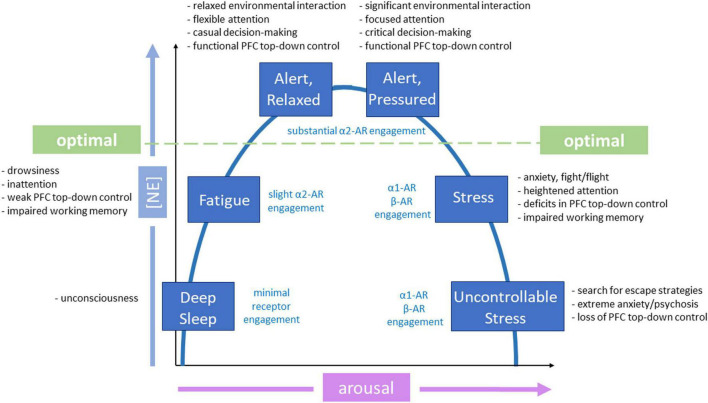
Inverted-U functionality of different adrenergic receptors in behaviors. Inverted-U model of norepinephrine (NE) modulation relates to activation of various levels and types of adrenergic receptors and phenotypes. Different adrenergic receptors are activated depending on level of arousal. The green dotted line indicates the threshold for optimal prefrontal functioning, influenced by substantial α2-adrenergic receptor (AR) engagement. Phenotypic manifestations associated with each state and level of NE release are also provided. Modified from Cools and Arnsten (2022).

The focus of NE-related neuroscience research in recent decades has centered on understanding how activation or inhibition of these adrenergic receptors may affect different behaviors and their clinical implications in the treatment of various neurological disorders. The functionality of neuromodulators, including NE, follow an inverted-U model (Figures 3, 4; Arnsten, 2011; Cools and Arnsten, 2022). Under normal conditions, NE provides essential regulation of the PFC to keep neurons in an “awakened” state where they can effectively process and exchange information with one another. When conditions vary from “normal,” *i.e.*, hypo- or hyper-arousal, NE-prefrontal dynamics also change. This dose-specific model demonstrates that during the moderate release of NE, PFC functioning is strengthened and sculpted to optimize function based on environmental demands; this results in alert phenotypes with optimal working memory, cognition, and attentional control. Conversely, in situations where NE release is either too sparse or too intense, a hindrance to PFC functioning occurs, and behavioral impairments arise (Arnsten, 2009, 2011; Chandler et al., 2014b; Xing et al., 2016; Northoff and Tumati, 2019; Ross and Van Bockstaele, 2021). For example, too little NE results in drowsiness and hypo-vigilance; contrastingly, too much NE elicits symptoms such as hyperarousal and anxiety. Furthermore, the varied release of NE into the PFC can cause differential receptor activation and consequent control of decision-making, arousal, and attention. Thus, the inverted-U model provides a basis for understanding how varying amounts of NE release influences prefrontal top-down control over other brain regions. Given the association between anxiety and excessive NE release, here we focus more on each adrenergic receptor subtype and its role in hyperarousal; hypoarousal, the other end of the inverted-U, is also briefly discussed (Figure 4).

### Hyperarousal: NE overload

#### α1 adrenergic receptors

Excessive NE release, as would occur during an environmental stressor, activates lower affinity α1 adrenergic receptors (Arnsten et al., 1998; Arnsten, 2009; Datta et al., 2019). Bulk activation of these receptors depletes functional connectivity to more regulatory parts of the brain involved in executive function (such as the PFC), while enhancing connections to brain regions involved in emotional processing. This impairs higher-order functional abilities of the PFC, such as working memory and attention, and shifts the brain from a state of top-down control (PFC-mediated, thoughtful control) to bottom-up control (salience-driven, impulsive control that is mediated by subcortical structures). Specifically, α1-AR activation releases Ca^2+^ from intracellular stores through the PLC-PKC pathway (Ramos and Arnsten, 2007). Once this system is activated and the animal is aroused, it is difficult to deactivate this system, as it engages physiological processes that are designed to directly aid in the survival of an organism (Moran, 2016). Resulting arousal acts in a positive feedback loop, as NE neurons change their firing rate by arousal state (Arnsten, 2009). Thus, as α1 receptors are activated in the PFC, arousal state increases, excessive NE release is prolonged, and α1 receptors continue to be activated. Activation of α1 receptors is accompanied by intermediate levels of both tonic and phasic firing in the LC (Atzori et al., 2016).

Activation of prefrontal α1-ARs via phenylephrine, an α1 agonist, resulted in impaired working memory performance in spatial alternation tasks in rats. This effect was rescued through administration of the α1 antagonist, urapidil (Arnsten et al., 1999; Birnbaum et al., 1999). Pharmacological studies utilize α1 receptor blockers, such as prazosin, to treat hyper-arousal related symptoms of post-traumatic stress disorder (PTSD) (Arnsten, 2007). In addition, the neuroleptic α1 blocker, clozapine, prevented stress-induced impairments of cognitive functioning in rodents and non-human primates (Arnsten, 1998), further connecting the overactivation of these receptors to hyperarousal. The anxiogenic effects of α1-AR activation and anxiolytic effects of α1-AR blockade support the idea that activation of these receptors promote hyperarousal states that may lead to prefrontal dysfunction.

#### α2 adrenergic receptors

The α2-AR agonist guanfacine enhances prefrontal cortical functions in rats, monkeys, and human beings and ameliorates prefrontal cortical deficits in patients with ADHD (Franowicz et al., 2002; Carr et al., 2007; Wang et al., 2007). Blockade of α2 receptors in the primate PFC erodes delay-related firing and instigates a variety of symptoms of ADHD, including limited impulse control and impaired working memory, leading to increased distractibility (Arnsten, 2007, 2009; Gamo et al., 2010; Ross and Van Bockstaele, 2021). Over-induced NE release facilitates the engagement of α1 receptors and reduces the beneficial cognitive control provided by α2-AR activation (Arnsten, 2000, 2009). It is likely that under conditions of hyperarousal and excessive release of NE, α2 receptors completely lose their beneficiary effect on prefrontal function and are overpowered by the activation of α1 and β receptors.

Psychostimulants such as amphetamine and methylphenidate are given in low doses to enhance the release and prevent the reuptake of NE in the PFC (Berridge et al., 2006). These drugs given in small doses emphasize the fine line of NE between beneficial α2 stimulation and detrimental α2 receptor inactivation (Arnsten, 2007).

#### β adrenergic receptors

Excessive NE release engages β-ARs. This activation is associated with fight-or-flight response, life-or-death decision-making, high limbic activation, and likely impairment of PFC functioning. Massive engagement of cortical and subcortical β-ARs results in deficits in working memory and favors impulsive and autonomic sympathetic responses (Bouret and Sara, 2005; Hains and Arnsten, 2008). Hyperarousal and high β-AR engagement are accompanied by maximum levels of LC tonic firing and low levels of phasic firing (Atzori et al., 2016). Though intermediate levels of LC tonic firing can be helpful for normal attentional functioning, high tonic firing has been associated with anxious states (Morris et al., 2020), as well as distress and neurodegeneration (Atzori et al., 2016). Supporting this claim, β agonists induced anxiogenic effects in rodents (Hecht et al., 2014). Further, β activation impairs fear extinction (Atzori et al., 2016) and remote footshock-induced memory (Fan et al., 2022), which may lead to a more dramatic and persistent anxiogenic response upon bulk activation. Though research surrounding specific β-AR subtype modulation in the PFC is sparse, a recent study demonstrated β2 optogenetic activation in the mPFC resulted anxiogenic responses in the OFT and EZM. These effects were attenuated through miRNA knockout of β2 mPFC pyramidal cell receptors (Lei et al., 2022).

The use of β blockers rescues attenuation dopaminergic modulatory effects following acute restraint stress (Chang and Grace, 2013). Specifically, administration of propranolol, a β-AR antagonist, restored DA function through reversal of stress-induced attenuation of VTA dopamine neuron population activity. Moreover, β antagonism has ben shown to prevent the development of anxiety-like behaviors in mice (Gorman and Dunn, 1993) and humans (Jefferson, 1974) through modulation of anxiety-related somatic responses (Hayes and Schulz, 1987). In several other studies, administering β blockers decreased the biochemical and behavioral effects of social stress (Wohleb et al., 2011), restraint stress (Tamburella et al., 2010), and shock-probe defensive burying response (Bondi et al., 2007). In addition, administration of the β1 antagonist, betaxolol, improved working memory in both rats and monkeys, suggesting blockage of these receptors improves prefrontal cognitive functioning (Ramos et al., 2005). The anxiolytic effects of the β blockers support the notion that these receptors likely contribute to hyperarousal following excessive NE release.

### Hypoarousal: insufficient NE

#### α1 adrenergic receptors

Generally, α1 receptors activate neurons to promote wakefulness and sustain neuronal activity. Insufficient stimulation of these receptors through inadequate NE release is less likely to be detrimental to cognition, as in the case of overstimulation, but likely induces inactivity and fatigue (Atzori et al., 2016). Additionally, given the lower binding affinity of α1 receptors compared to α2-ARs (Ramos and Arnsten, 2007), it is possible that very low levels of NE do not engage α1-ARs to cause detrimental behavioral phenotypes.

#### α2 adrenergic receptors

Since α2-ARs play a crucial role in optimal PFC functioning, insufficient activation of α2 receptors primarily impacts cognition and attention. Constant hypoactivation of α2 receptors may lead to impaired prefrontal, subcortical, and motor functioning, representing a depressive state. In human postmortem studies of patients diagnosed with major depressive disorder (MDD), increased α2-agonist ligand binding was observed at α2-adrenegic autoreceptors on NE neuronal cell bodies (García-Sevilla et al., 1999; Hamon and Blier, 2013). Consistently, postmortem analyses of the PFC of MDD-diagnosed suicide victims showed increases in mRNA levels of presynaptic inhibitory α2 autoreceptors (Escribá et al., 2004). Moreover, low stimulation of postsynaptic prefrontal α2 receptors induces symptoms such as cognitive impairment, inattention, and drowsiness (Blier and Briley, 2011). These findings suggest that insufficient levels of noradrenergic neurotransmission may contribute to depression etiology.

#### β adrenergic receptors

Insufficient activation of β-ARs, given their role in anxiogenesis, may not be as detrimental to prefrontal functioning as hypoactivation of other adrenergic receptors. Interestingly, down-regulation of beta adrenergic expression has been observed with antidepressant treatment (Stahl, 1992). Further, given that β-ARs have the lowest binding affinity of all noradrenergic receptor subtypes (Ramos and Arnsten, 2007), it is possible that lack of engagement of these receptors does not induce problematic or observable phenotypes.

## Prefrontal circuit-level top-down modulation of anxiety

**FIGURE 5:**
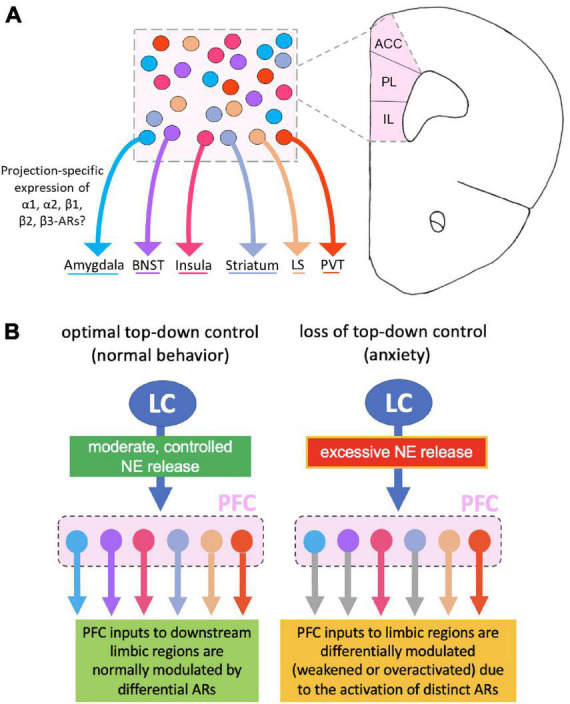
Norepinephrine (NE) modulation of distinct prefrontal cortical neurons that project to different anxiety-related subcortical regions. **(A)** The prefrontal cortex (PFC) sends dense projections to numerous brain regions implicated in anxiety, yet there is currently no research on which NE receptor subtypes exist on these output pathways. **(B)** Left panel: Potential circuit-based mechanisms by which optimal locus coeruleus (LC)-mediated NE release in the PFC modulates different PFC output circuits through activation of differential adrenergic receptors (ARs). Right panel: Excessive NE release weakens or over activates PFC outputs to the downstream limbic regions, depending on projection-specific activation of distinct AR subtypes. This in turn results in a shift of dynamic balance among the different circuitries, leading to a loss of prefrontal top-down control and the production of an anxiety-like phenotype.

The execution of anxiety-related behaviors involves detection of environmental stimuli through sensory systems, assignment of emotional value to these cues via subcortical structures, and execution of behavior based on this information via cortical modulation. The PFC is thought to coordinate situational evaluation and corresponding behavioral outcomes through its extensive connections with other regions of the brain, including the amygdala, bed nucleus stria terminalis (BNST), insula, striatum, lateral septum, and the paraventricular thalamic nucleus (PVT), among others in underlying anxiety circuits (Calhoon and Tye, 2015; Mack et al., 2022; Figure 5). Elucidating distinct neural circuit dynamics involved in PFC control of maladaptive behaviors in pathological anxiety can provide insight into the neural pathology underlying dysfunction and may provide an avenue for future circuit-based treatments. Circuit-level modulation is often conserved in translation from mouse to human (Calhoon and Tye, 2015; Poe et al., 2020; Anastasiades and Carter, 2021), allowing for experimentation with animal models that offer clinical applications within this sector of modern neuroscience.

Given what is known about the impact of varying levels of NE on prefrontal function, it is likely that these modulatory effects alter the activity of PFC projection neurons regulating downstream anxiety-related brain regions. To fully understand how NE modulates top-down prefrontal control, a better understanding of NE modulation of distinct PFC connections is necessary. Though very little is known about NE actions on unique PFC circuits, understanding the important prefrontal circuits involved in anxiolytic responses provides a direction for targeted investigation of how NE specifically modulates these pathways and affects consequent anxiety-related behaviors. In this section, we emphasize what is currently known about PFC, pathological anxiety and avoidance behavior through distinct PFC top-down connections to anxiety-relevant brain regions. This provides a basis for our later proposed model that integrates NE into the prefrontal top-down modulation of anxiety.

### PFC-amygdala

The amygdala is one of the most highly studied brain regions regarding mood and anxiety disorders, and has long been investigated for its direct role in regulating sustained anxiety symptoms (Tye et al., 2011; Felix-Ortiz et al., 2013, 2016; Allsop et al., 2014; Felix-Ortiz and Tye, 2014; Calhoon and Tye, 2015). This brain region plays a significant role in emotional processing, cognitive evaluation of emotional stimuli, and emotional learning through its intricate connections to cortical and subcortical regions (Janak and Tye, 2015; Giovanniello et al., 2020). The amygdala is made up of several subnuclei [basolateral amygdala (BLA), central amygdala (CeA), centromedial amygdala (CeM), and basomedial amygdala (BMA), among others] (Marek et al., 2019), whose contribution to anxiety differs depending on the subregion (Treit et al., 1993; Möller et al., 1997; Moreira et al., 2007). The PFC sends projections to various subregions of the amygdala, including the BLA and CeA (Coley et al., 2021). Activation of the entire PFC produced a reduction in amygdala activity, demonstrating the inhibitory effect of PFC projections to the amygdala (Quirk et al., 2003). Moreover, specific effects of prefrontal regulation of the amygdala often vary by PFC subregion.

Both the prelimbic (PL) and infralimbic (IL) subregions of the rodent PFC project to BLA and CeA subregions. In a study by Adhikari et al. (2015), activation of the IL projections to the BMA resulted in reduced anxiety-like behaviors and physiological responses. In contrast to the anxiolytic effects of IL inputs to the amygdala, connections from the PL subregion are thought to drive fear expression (Pendyam et al., 2013; Marek et al., 2019) and promote anxiety (Kim et al., 2011). Haikonen et al. (2022) recently combined viral tracing and electrophysiological techniques to examine the effects of maternal separation (MS) on mPFC-to-BLA connectivity and function in young (P14-21) rats. Prolonged MS as an early-life stressor in young rodents is thought to induce emotional and behavioral abnormalities in adulthood, including increased anxiety-like behavior (Kestering-Ferreira et al., 2021). Interestingly, mice that underwent this MS-induced anxiogenic protocol demonstrated increased prefrontal inputs to BLA GABAergic interneurons and a transient increase in the strength of feed-forward inhibition in the BLA during development. The enhanced GABAergic inhibition raises the induction threshold of long-term potentiation and associates with lower functional synchronization within prefrontal-amygdala networks *in vivo*. These changes are sex-dependent, with the parameters detected in male but not female rats, who were also resistant to MS-dependent changes in anxiety-like behaviors (Haikonen et al., 2022).

In human studies of clinical anxiety disorders, consistent hyperactivation of the amygdala was observed (Etkin and Wager, 2007; Boehme et al., 2014). In fact, in early human connectivity analyses, subjects with more anxious temperaments had reduced vmPFC-amygdala coupling when presented with aversive stimuli (Pezawas et al., 2005). Interestingly, though varying from mouse studies, the dlPFC (dorsolateral PFC, analogous to rodent PL) exerted a significant inhibitory influence on the right amygdala that was absent in patients with GAD (Dong et al., 2019). Further, in patients with SAD (social anxiety disorder), decreased activity in PFC was also observed (Martin et al., 2010), which may explain some of the cognitive deficit-related symptoms observed in anxiety disorders. It is posited that overactivation of the amygdala observed in anxiety disorders is driven by the loss of prefrontal top-down control. This claim is supported by a recent study that found that stronger vmPFC-amygdala connectivity predicted lower anxiety levels (Kim and Whalen, 2009; Kim et al., 2011). Further, strength of dlPFC-amygdala connections were also correlated with anxiety levels, with the least anxious individuals having the most robust connections (Etkin et al., 2009). Given this evidence, it is likely that the loss of PFC-regulated top-down control is implicated in amygdala-mediated anxiogenic responses.

Hypoactivation of the PFC may lead to hyper-responsivity of the amygdala to even non-threatening environmental cues, triggering full-scale responses often observed in PTSD (Whalen et al., 1998), panic disorder (Kent and Rauch, 2003), and other anxiety disorders. This idea is displayed in mouse models, as mice with limited prefrontal-amygdala interaction displayed significant threat-generalization (Charney et al., 2017). Altogether, the evidence summarized here supports an association between PFC-amygdala circuitry and anxiety. Despite this, it remains unknown whether and how NE may modulate PFC projections to the amygdala under both normal and pathological conditions. While it has been shown that NE release in the BLA promotes anxiety-like behavior (McCall et al., 2017), it remains unclear how NE release in the PFC may act on BLA-projecting neurons. Given the inverse relationship between PFC-amygdala connectivity and anxiety, it is plausible that excessive NE release disrupts functionality of this circuit through loss of prefrontal top-down control.

### PFC-BNST

Though the bed nucleus stria terminalis (BNST) is a relatively small brain region, it can be divided into 18 heterogeneous subregions (Robinson et al., 2019). These subregions are seemingly distinct and, at times, opposing in functionality (Jennings et al., 2013; Kim et al., 2013). Previous rodent studies have demonstrated direct input from the PFC to the BNST (Dong et al., 2001; Vertes, 2004; Radley and Sawchenko, 2011; Radley et al., 2013; Glangetas et al., 2017; Johnson et al., 2019) that is particularly dense in IL-avBNST circuits but is also present between the PL and avBNST regions (Johnson et al., 2019). Rodent studies investigating vmPFC-BNST and ACC-BNST (anterior cingulate cortex-BNST) connections in anxiety-like behaviors found that animals exposed to shock demonstrated decreased connectivity in both circuits (Alvarez et al., 2011). This finding supports a relationship between anxiety-like behaviors and loss of PFC top-down modulation of the BNST in rodents.

Moreover, silencing of PL inputs to the avBNST with optogenetics resulted in anxiogenic behavioral phenotypes, including increased immobility and elevated hormonal stress responses during shock-probe burying and tail suspension tests (Radley et al., 2009; Johnson et al., 2019). These results suggest a functional role of PL inputs to the avBNST in reducing anxiety-like behavior. Contrastingly, in a backward conditioning paradigm, IL inputs to the BNST are activated by unpredictable threats (Goode et al., 2019). This may have translational applications to anxiety- and other stress-related disorders, where threats are often inexplicit and unpredictable.

Evidence of prefrontal innervations to the BNST also exist in humans (Dong et al., 2001; Vertes, 2004; Motzkin et al., 2015). Though not PFC-BNST circuit specific, meta-analyses of neuroimaging studies have demonstrated heightened activation of the BNST while subjects awaited aversive cues (Clauss et al., 2015). Further, a correlation between level of BNST activation and arousal during exposure to unpredictable shocks was observed (Alvarez et al., 2011). In patients with PTSD, heightened sensitivity to stimuli, a hallmark of anxiety disorders, is associated with increased BNST activation. Moreover, increased BNST activity was observed in patients with GAD when compared to healthy controls (Yassa et al., 2012). Thus, there are several lines of evidence that support BNST-based modulation of hypervigilance and hyperarousal. It is possible that in the presence of a stressful or aversive event, BNST activation modulates a sustained, or continuous, anxious state. This heightened state may be exaggerated in anxious individuals (Yassa et al., 2012), resulting in some of the physical and emotional symptoms exhibited in anxiety disorders.

Given what is known about the anatomical and functional heterogeneity of PFC subregions, varying efferent connections from different cortical subregions to the BNST likely control different aspects of anxiety-like behavior (Jennings et al., 2013; Kim et al., 2013). Though similar anxiety-inducing experiments have not been conducted in humans due to ethical and technical limitations, considering the homology that exists between the rodent and human brain, similar anxiety-specific changes in PFC-BNST circuitry are likely present. The studies discussed above provide a robust connection between PFC-BNST modulation and anxiety disorders, but the downstream circuitry, cell-type specificity, and subregion modulation of these symptoms remain elusive. However, increased activity of the BNST and decreases in PFC-BNST connection during anxiety-like behavior supports the notion that anxiety may be due to loss of PFC top-down control to the BNST.

### PFC-insula

The insula plays an essential role in emotional experience and subjective feelings (Calder et al., 2000; Borg et al., 2013), making it an essential node in the anxiety network. Given its extensive connections with the amygdala and PFC (Paulus, 2008; Simmons et al., 2008; Engel et al., 2009; Freese and Amaral, 2009), the insula has been consistently implicated in the etiology of anxiety disorders (Damsa et al., 2009). This brain structure is divided into rostral agranular insular cortex (raIC); gustory insular cortex (gIC); primary interoceptive posterior insular cortex (pIC), and caudal insular cortex (cIC) (Bruce et al., 2012). Chemogenetic experiments have revealed insular subregions have distinct and often opposing roles in anxiety response. For example, rostral regions play an anxiogenic role (as raIC inactivation increased exploratory behavior and activation decreased these behaviors), whereas caudal regions produce anxiolytic responses in rodents (cIC inactivation decreased exploration and cIC activation promoted exploratory behavior, indicative of decreased anxiety). Conversely, activation of raIC and gIC induced opposite anxiogenic effects, confirming prior results (Bruce et al., 2012).

In a human study by van Tol et al. (2012), subjects were given a word-encoding task (where subjects were presented with positive, negative, or neutral words) and found that negative words had greater insular activation in patients with anxiety disorders compared to healthy controls. Moreover, healthy patients with greater trait anxiety levels had proportional increases in insular activation (Stein et al., 2007), showing anxiety-like symptoms recruit activation of the insula. In healthy subjects, increased levels of trait anxiety consequently resulted in increased insular activation (Engel et al., 2009). Similarly, the degree of insular (right middle insula) activation in women diagnosed with PTSD was greater than in trauma-exposed controls (Lanius et al., 2007; Lindauer et al., 2008; Strigo et al., 2010). This pattern of heightened insular activation in patients with PSTD was further observed when subjects were exposed to emotional, trauma-unrelated stimuli (Simmons et al., 2008; Fonzo et al., 2010). Patients with anxiety disorders may constantly entertain exaggerated interoceptive cues generated by the overactivated insula, which could increase anxiety symptoms and lead to further elevation of insular activity (Stein et al., 2007).

Hyperactivation of various brain regions in anxiety disorders is thought to be attributed to loss of top-down control via vPFC hypoactivation (Bruce et al., 2012). Though PFC-IC circuit-specific research is limited, hypoactivation of the PFC is related to emotional control in patients with PTSD (Etkin and Wager, 2007). Further, decreased ACC volumes were positively correlated to the presence of PTSD symptoms (Kasai et al., 2008). This evidence combined with increased insular activity in these disorders supports the idea that the relationship between increased insular activation and anxiety is due to a loss of PFC top-down control to the insula. With simultaneous increased insular activation and loss of top-down control from the PFC, this circuit may serve in the development or exacerbation of anxiety disorders.

### PFC-striatum

The striatum is a complex brain region that contributes to a plethora of behavioral processing implicated in anxiety disorders, including attention, motivation, and learning (Lago et al., 2017). Prior studies investigating the role of the striatum in anxiety disorders often focus on the ventral striatum for its role in processing affect (Cardinal et al., 2002; Christakou et al., 2004; Schott et al., 2008) but the dorsomedial (dmS) striatum has been and found to influence other aspects involved in anxiety disorders, including decision making (Balleine et al., 2007), avoidance behavior (Aupperle and Paulus, 2010; Aupperle et al., 2015; LeBlanc et al., 2020), and action initiation (Porter et al., 2015). Interestingly, deep brain stimulation of the striatum in rodents (Rodriguez-Romaguera et al., 2012) and humans (Rauch et al., 2006; Denys et al., 2010) has shown that activation of this brain region results in a reduction in anxiety-related symptoms.

In rodents, an especially relevant input to the dmS is the dorsomedial PFC (Sesack et al., 1989; Gabbott et al., 2005). The role of this dmPFC-dmS circuit has previously been demonstrated in decision-making under conflict, a trait that is often disrupted in anxiety disorders (Friedman et al., 2015). A recent study reported that greater activity in dmPFC-dmS projection neurons was observed during open arm occupancy compared to that of the closed arms in the elevated plus maze (EPM); this effect was not observed in other dmPFC circuits, such as the dmPFC-amygdalar projection neurons. Further, stimulation of the dmPFC-dmS pathway increased open arm exploration, showing an increased drive to approach and decreased anxiety-like behavior (Loewke et al., 2020). Inhibition of this circuit decreased open-arm exploration, illustrating the involvement of this pathway in anxiety-related avoidance. These findings provide evidence supporting the control of dmPFC-dmS circuitry in regulating anxiety-like behavior in the EPM and elevated zero maze (EZM) (Loewke et al., 2020). Moreover, these results support the model of prefrontal top-down control over defensive action, such as avoidance. Investigators have posed that corticostriatal circuitry may integrate previous learning contingencies and the behavioral state of the organism to integrate signals and select subsequent appropriate behavioral responses. This hypothesis is supported by the cortical processing of aversive experience; it is reasonable to conclude that this circuit plays a key role in the generation of defensive response via prefrontal-striatal projection neurons (Kirouac, 2021).

Ventral and dorsomedial regions of the striatum receive prominent PFC afferents (Cisler and Koster, 2010; Liljeholm and O’Doherty, 2012; Calhoon and Tye, 2015; Howland et al., 2022). The ventral striatum is known to be involved in learning and motivation (Dayan and Balleine, 2002; O’Doherty et al., 2004; Cauda et al., 2011; Porter et al., 2015). Motivation is often thought of in the context of addiction (Martin et al., 2002; Chambers et al., 2003), but can be included in anxiety research when reframed to the context of a motivation to avoid danger, or risk avoidance (Lago et al., 2017). This is particularly relevant as individuals diagnosed with anxiety disorders tend to possess abnormally high risk-avoidance levels, which could be attributed to ventral striatal dysfunction (Lago et al., 2017). Regarding circuit-level connectivity, the ventral striatum, which includes the nucleus accumbens (NAc) shell and core, receives input from orbitofrontal and anterior cingulate cortices in humans (Heimer et al., 2007; Ernst and Fudge, 2009; Bolstad et al., 2013; Porter et al., 2015). The ventral striatum, especially NAc, receives dense excitatory afferents from the PFC. NAc volume appears to be a predictor of anxiety symptoms following treatment (Burkhouse et al., 2020), while NAc deep brain stimulation decreases ratings of depression and anxiety (Bewernick et al., 2010). Many noradrenergic dopamine-beta-hydroxylase immunoreactive (DBH-ir) fibers were found in the shell but few were in the core regions (Berridge et al., 1997). A further study indicated that the primary source of NE afferents to the shell of NAc is from the A2 region, with lesser contribution from the A1 and LC (Delfs et al., 1998). Thus, LC-mediated NE release may influence NAc activity through PFC projection neurons. However, how PFC-NAc pathway is modulated by NE and which receptor subtypes are involved in the regulation of anxiety-like behavior remain to be determined.

### PFC-PVT

The paraventricular thalamus (PVT) is a midline thalamic structure that integrates information from the motor, limbic, and cortical circuits in the brain (Sesack et al., 1989; Vertes, 2004; Gao et al., 2020, 2023; Iglesias and Flagel, 2021; Penzo and Gao, 2021). The PVT is often separated anatomically and functionally into two subregions: the anterior PVT (aPVT) and posterior PVT (pPVT) (Li and Kirouac, 2012; Kirouac, 2015). Robust sources of input to this brain region include the IL and PL cortices (Li and Kirouac, 2012; Kirouac, 2015), as indicated through retrograde (Krout et al., 2002; Otake et al., 2002) and anterograde (Hurley et al., 1991; Canteras and Swanson, 1992; Vertes, 2004) studies in rodents. The PVT has been known to be a key node in the emotional processing network (Barson et al., 2020) and mediates behavioral responses to stress.

The aPVT receives information from the IL concerning motivational state and arousal. The pPVT receives input from both PL and IL subregions of the PFC, which is thought to communicate information about salient emotional stimuli (Otis et al., 2017, 2019).

Due to the known involvement of the PVT and the PFC in fear, anxiety, and arousal, this circuit may work to modulate behavioral responses to aversive and/or threatening stimuli, though more work is needed to confirm this hypothesis. Although gaps in knowledge surrounding PFC-PVT circuitry do exist, the dense connections between these regions and the behaviors they are known to regulate suggest a likely top-down influence of the PFC on the PVT. While NE signaling in the PVT has been shown to influence cellular responses to stress (Beas et al., 2018), an interesting avenue of future research will be determining how NE release in the PFC alters activity between the PFC and the PVT, and how this may relate to stress and pathological anxiety.

### PFC-lateral septum

The lateral septum (LS) is a forebrain region implicated in various behaviors, including feeding, rewards, sociability, and fear. Alongside these functions, the LS has long been involved in the control of stress responses and anxiety (Sheehan et al., 2004). This brain region was once described as a homogenous structure, but has now been recognized as a heterogeneous region with different subregions, cell types, and microcircuits (Risold and Swanson, 1997). The LS can be characterized into four major subregions, dorsolateral septum (dlLS), dorsomedial septum (dmLS), ventrolateral septum (vlLS), and ventromedial septum (vmLS), each exhibiting differential effects on anxiety based on their unique afferent and efferent connections (Rizzi-Wise and Wang, 2021). For example, the dorsal LS is implicated in promoting anxiety (Thomas et al., 2013), while the activation of ventral LS regions reduces anxiety (Parfitt et al., 2017) and fear (Parfitt et al., 2017; Besnard et al., 2020). Phenotypes that arise from vLS activation suggest this region plays a role in suppressing negative affect (Rizzi-Wise and Wang, 2021), thus blunting the psychological severity of stressors (Sheehan et al., 2004). On the other hand, lesions to the LS produce “septal rage” or over-reactivity to stimuli and excessive fear response (Albert and Chew, 1980). Similarly, inhibition of individual LS neurons increases anxiety (Sheehan et al., 2004).

There have been significantly fewer studies investigating PFC inputs to the LS. The IL subregion of the PFC sends dense projections to the intermediate parts of the LS, moderate inputs to the dorsal LS, and sparse inputs to the ventral LS (Hurley et al., 1991; Canteras and Swanson, 1992; Vertes, 2004). One of the only studies investigating PFC modulation of the LS found that optogenetic activation of PFC terminals in the LS had overall anxiogenic effects, as shown by increased open arm avoidance and decreased open arm entry probability in the EPM, as well as increased freezing and decreased time spent in the center of the arena in the open field test (OFT) (Chen et al., 2021). Further, opto- and chemo-genetic inhibition of the IL-LSe pathway produces anxiolytic effects, as observed through decreased open arm avoidance, increased probability of open arm entry, and increased center occupancy in the OFT (Chen et al., 2021). These findings identify the LS as a key target of IL to enhance anxiety-related behavioral responses, suggesting a direct, local IL-LS synaptic connection to modulate anxiety and fear (Chen et al., 2021). However, this finding seems inconsistent with the idea of top-down prefrontal control of the proper behavioral response. Further studies in a IL-LS subcircuit- and cell-type-specific manner would provide novel insight into the role of IL-LS pathway in anxiety-like behaviors (Besnard and Leroy, 2022).

## A model of NE modulation of PFC top-down control

Despite the well-known sensitivity of the PFC to changes in the LC-NE system (Arnsten, 1998), it remains almost completely unexplored how NE release differentially influences each of the PFC circuits mentioned above. Especially, if these distinct PFC projections express different adrenergic receptors and whether they are differentially modulated by LC activity and its released NE in the PFC (Figure 5). Nonetheless, some evidence suggests NE regulates distinct PFC populations. For example, adrenergic modulation shifts the dynamic properties of corticopontine (CPn) but not commissural (COM) neurons and increases the excitability of CPn neurons significantly more than COM neurons (Dembrow et al., 2010), indicating subcircuit-specific neuromodulation in the PFC. These findings describe some of the functional consequences of selective neuromodulation on behavioral states during goal-directed behavior (Dembrow and Johnston, 2014). Evidence of differential effects of NE signaling on varying subcircuit-specific PFC modulation, though limited, inspires the idea that other prefrontal circuits are uniquely modulated by NE in the PFC. Combining our knowledge of PFC anxiety-related circuits with the molecular and behavioral framework of general NE modulation in the PFC, we propose a model of adrenergic influence on prefrontal top-down control of anxiety (Figure 5).

In this model, we posit that controlled NE release in the PFC maintains optimal functioning of the PFC, eliciting control over other more emotionally-related limbic regions involved in anxiety (eg., amygdala, BNST, insula, striatum, PVT). We pose that this prefrontal top-down control integrates limbic responses with conscious planning and decision-making to elicit appropriate behavioral responses (Figure 5). Conversely, conditions of excessive NE release, evoked by unpredictable environmental threats or other perceived psychological stressors (such as those observed in anxiety disorders), may either weaken or overactivate PFC projections to these anxiety-related brain regions. Given that excessive stimulation of ARs inhibits cognitive functioning (Arnsten, 1998) and may take the PFC “offline,” it is likely that the loss of top-down control over some or all of these aforementioned brain regions would shift brain states to a mode of subcortical modulation and thus play a vital role in the generation of anxiety (Figure 5).

However, this model has yet to be tested directly, and understanding exactly which PFC circuits are impacted by excessive NE release is of great interest from a preclinical and clinical perspective. Altogether, there is a need for more research to elucidate the impact of NE on specific PFC circuits known to be involved in pathological anxiety.

It is also noteworthy to consider the reciprocal connections between the PFC and each or some of these brain regions, and how these regions may individually impact optimal prefrontal functioning in the context of NE signaling. While it remains unknown which AR receptors are expressed on specific PFC output populations, it is also unclear whether afferent inputs to the PFC from other anxiety-related brain regions (*e.g.*, ventral hippocampus) also express AR receptors, which could further contribute to substantial modulation of PFC activity following NE release. Although PFC projection pathways have been the focus of this review, the influence of NE on distinct afferent inputs proves to be a critical area of research in circuit- and systems-based neuroscience. More work is needed to understand whether and how PFC inputs and outputs are uniquely engaged by NE to regulate anxiety-like behaviors.

Finally, it should be noted that given the reciprocal descending pathways from the PFC to the LC, any dynamic activity changes in the mPFC could also have a feedback effect on the LC neuronal activity. However, there are limited studies investigating prefrontal-LC projections, making the speculation of how these projections may affect anxiety-like behavior challenging. Nevertheless, it was reported that electrical stimulation of the PFC in anesthetized rats activated the LC through NMDA and non-NMDA mechanisms (Jodo and Aston-Jones, 1997). Given the PFC’s role in attention via NE modulation (Arnsten, 1998, 2011; [Bibr B34][Bibr B166]), presumably it is possible that low level’s of NE in the PFC can induce activation of the LC, which increases NE release to an optimal level to regulate sustained attention and decision-making. In contrast, a high level of NE release in the PFC, as would occur during stress or anxiety-evoking situations, can disengage optimal prefrontal functioning. Therefore, it is unlikely these prefrontal projections to the LC provide negative-feedback signals to provide top-down control to the LC to inhibit NE release during anxiety-like behavior. Even so, more research is needed on the neurochemical and behavioral effects of this descending pathway on anxiety-like behaviors.

## Future directions

Preclinical experiments suggest that all NE receptor subtypes participate in anxiety-like processes. Given the extensive use of pharmacological agents that target NE receptors to treat pathological anxiety and the clear relationship between PFC dysfunction in the clinical population, revealing the intricacies of NE receptor signaling in modulation of PFC circuit-level control of anxiety is crucial.

Overall, evidence supports a decisive role for NE and distinct PFC circuits in driving anxiety-related behaviors. Significant progress has been made in the last two decades in investigating the LC-NE system’s direct influence on anxiety and other aversive behaviors. Despite these advances, more work is needed to bridge the gap between NE signaling and PFC circuit function in anxiety by revealing the precise circuit-level effects of NE release in the PFC. Although the field has yet to directly investigate NE-PFC influences on anxiety etiology, asking questions from a combinatorial standpoint of molecular signaling in conjunction with known circuit-based regulation of behavior, as in our proposed model, is now possible due to recent technological advances. For instance, particularly in rodents, new tools have provided extraordinary temporal and spatial resolution to investigate causal functions of neural circuits and have already yielded impressive results identifying discrete PFC circuits mediating specific anxious behaviors, including approach-avoidance, social deficits, and fear. Our model, though speculatory, can begin to be directly tested using tools such as the GRAB-NE biosensor to detect endogenous NE release in the brain, offering an unprecedented opportunity to uncover the temporal dynamics of NE signaling in the PFC and its resulting effects on behavior (Feng et al., 2019). These dynamics can be fine-tuned even further using the Cre-Lox system to restrict NE biosensor expression to specific PFC circuits and excitatory versus inhibitory PFC cell types. In addition, increasingly advanced computational data analysis, such as deep learning to analyze micro-behaviors, may reveal additional anxiety-like behaviors in rodents that were previously overlooked by manual scoring. Moreover, retrograde tracing studies to visualize exactly where NE-modulated PFC-projection neurons are located, in conjunction with fluorescent *in situ* hybridization (FISH) for specific NE receptor subtypes will further pry into the unknown details of NE receptor expression on distinct PFC-circuits. With vigorous investigation using these new tools, we can begin to address our proposed model and many other outstanding questions that remain (see Box 2–Outstanding questions). Implementing a combination of these new and improved techniques is needed to truly uncover the precise dynamics of the LC-NE-PFC’s role in anxiety, which is an exciting destination in the future of neuroscience research.

**BOX 2 Outstanding questions.**

1. NE is known to modulate decision making and cognitive functions in the PFC, but the circuit-level mechanisms have been unexplored. Revealing which specific cell types and circuitry are affected by NE release in the PFC is an important and interesting avenue of future research

2. Sex differences are known to play a role in NE signaling and receptor expression in the PFC and related circuitry. Whether and how these sex differences contribute to the susceptibility or etiology of pathological anxiety still remains unknown.

3. PFC-NE plays a role in the functionality of the PFC and its ability to provide top-down control, yet the precise projection neurons and interneurons involved remains elusive.

4. The presence of α1 and α2 receptors have been identified in both excitatory and GABAergic neurons in the PFC. However, it is unknown if these receptors are colocalized on the same cells or if cells are constricted to receptor subtype specificity. Moreover, which adrenergic receptor subtypes are expressed on various PFC projection neurons, especially those efferent pathways known to be involved in the modulation of anxiety.

5. It is still unknown whether and how the development of pathological anxiety disorders alters NE release, adrenergic receptor expression, and/or sensitivity in PFC neurons.

In addition, though sex-differences in anxiety etiology is not discussed in this review, this should not be overlooked. The US National Institute of Mental Health reports that the lifetime prevalence of anxiety disorders is two to three times higher in women than in men (Yonkers et al., 2003; Grant et al., 2005; Kessler et al., 2006, 2009, 2012; Tolin and Foa, 2006; Leach et al., 2008) and women demonstrate distinctly lower treatment efficacy (Donner and Lowry, 2013). Whether and how sex hormones converge with NE signaling in the PFC to guide behavior, and how this may potentially mediate sex differences in pathological anxiety is another intriguing and important line of future research. Despite some recent progress, there is still a substantial delay in the conceptual idea that the field must study both males and females to effectively investigate and treat disorders across sexes. A future of inclusive data collection generates hope for filling the gap in knowledge involving the female brain and developing improved, comprehensive treatments for anxiety and other psychiatric disorders.

It is interesting to speculate that a particular neural circuit dysfunction could be casually involved in multiple psychiatric diseases, including anxiety. Further, given the substantial rate of co-morbidity and shared symptomology among various mental illnesses, the identification of distinct neurobiological mechanisms underlying these diseases is one of the most pressing needs and invigorating avenues of research into psychiatric disorders. Treating psychiatric disorders that disrupt these complex, intertwined neural systems may require a broad, circuit-level approach. A shift in how we consider the underpinnings of anxiety—such that the brain works in a circuit-dependent manner, where changes (including neuromodulatory influences) in each subregion affect the next—promises to remodel how anxiety disorders are treated.

Current technological advances for neuroscience experiments, particularly in rodents, have provided exceptional temporal and spatial resolution to investigate causal functions of neural circuits mediating specific anxious behaviors, including approach-avoidance, social deficits, and fear. Combinatorial approaches with increasingly advanced computational data analysis, such as deep learning to analyze micro-behaviors, will directly aid researchers in answering these critical questions. In particular, the recent advent of biosensors to detect endogenous NE release in the brain offers an unprecedented opportunity to uncover the temporal dynamics of NE signaling in the PFC and its resulting effects on the behavior. In particular, using the Cre-Lox system to restrict NE biosensor expression to specific PFC circuits and cell types, we can begin to address some of the outstanding questions that remain (Box 2). Implementing a combination of these new and improved techniques is needed to truly uncover the precise dynamics of the LC-NE-PFC’s role in anxiety, which is an exciting destination in the future of neuroscience research.

## Author contributions

NB, NM, and W-JG wrote the manuscript. All authors contributed to the article and approved the submitted version.
